# Systematic Review of Meta-Analyses: Exercise Effects on Depression in Children and Adolescents

**DOI:** 10.3389/fpsyt.2020.00081

**Published:** 2020-03-06

**Authors:** Mirko Wegner, Sandra Amatriain-Fernández, Andrea Kaulitzky, Eric Murillo-Rodriguez, Sergio Machado, Henning Budde

**Affiliations:** ^1^ Institute of Sport Science, Humboldt-Universität zu Berlin, Berlin, Germany; ^2^ Faculty of Sport Sciences and Physical Education, University of A Coruña, A Coruña, Spain; ^3^ Faculty of Human Sciences, Department of Pedagogy, Medical School Hamburg, Hamburg, Germany; ^4^ School of Medicine, Anahuac Mayab University, Mérida, Yucatán, Mexico; ^5^ Laboratory of Physical Activity Neuroscience, Physical Activity Sciences Postgraduate Program, Salgado de Oliveira University, Niterói, Brazil

**Keywords:** depression, physical exercise, intervention, children, adolescents

## Abstract

**Background:**

Depression is a common threat to children and adolescents in terms of affecting psychosocial development and increasing their risk of suicide. Apart from conventional treatments for depression, physical exercise has become a promising alternative. This paper aims to systematically review the existing meta-analyses that focus on the impact of physical exercise on clinical and nonclinical depression in children and adolescents.

**Methods:**

A systematic literature search was conducted using PsycINFO, PsycARTICLES, MedLine, PubMed, and hand searching. Risk of bias analysis, effect sizes calculations, and evaluation of the methodological characteristics (AMSTAR 2) were carried out.

**Results:**

Four meta-analyses met the inclusion criteria. After analysing the overlap, the total sample contained 30 single studies (mostly including gender mixed samples) and 2,110 participants (age range 5–20 years). The medium duration of the interventions was 11.5 weeks. The sessions had a medium length of 41 min, and the frequency of implementation was three sessions per week. The most implemented intervention type was aerobic exercise, while control groups mainly continued with their regular routine, among other related options. The overall mean effect of physical exercise on depression was medium (*d* = −0.50). The additional analysis in clinically depressed samples documented a small to medium mean effect (*d* = −0.48) in favor of the intervention.

**Conclusion:**

The small to medium but consistently positive effects that were found in the present study place physical exercise as a promising and helpful alternative for children and adolescents with clinical and nonclinical depression. The limited literature focused on children and adolescents in comparison with adult samples points to the need for further research.

## Introduction

Depressive symptoms and clinically relevant depressive disorders are a common threat to the mental health of children and adolescents (1). Depression is the leading cause of several diseases and disabilities in these age groups, which is why research on this topic should be intensified (2). Depression has multiple levels of severity (mild, moderate, or severe). It may appear as a single symptom of sadness, dejected mood or a complex of other symptoms described below. Depression in a nosological sense is diagnosed when a specific combination of symptoms occur over a definite period of time and with a particular intensity (3). The DSM-V (Diagnostic and Statistical Manual of Mental Disorders) defines major depression as having five or more symptoms over a period of two weeks (4). Symptoms include depressed or irritable mood, diminished interest or pleasure, insomnia or hypersomnia, psychomotor agitation or retardation, fatigue or loss of energy, feelings of worthlessness or inappropriate guilt, diminished ability to concentrate, recurrent thoughts of death, suicidal ideation with or without a specific plan or a suicide attempt. A persistent depressive disorder (dysthymia) is diagnosed when depressive symptoms are present for most days over at least one year. Compared to major depression, the symptoms are milder (4).

Common interventions against depression are pharmacological treatments and psychotherapy (5). Selective serotonin reuptake inhibitors (SSRI) are common interventions for clinical depression, however, side effects like weight gain, increases in blood pressure, and impairment of sexual functions are experienced (6). Furthermore, the effectiveness of antidepressants was questioned by placebo-controlled clinical trials showing only a small effect size (7). Psychological and pharmacological therapies had similar efficacies in the treatment of depressive disorders (8). However, active medication had a small but significant contribution to the overall efficacy of combined treatments (9). One review directly compared typical treatments using seven meta-analyses (with a total of 53 studies) for seven major types of psychological treatment for mild to moderate adult depression (cognitive-behavior therapy, nondirective supportive treatment, behavioral activation treatment, psychodynamic treatment, problem-solving therapy, interpersonal psychotherapy, and social skills training). There was no indication that one of the treatments was more or less efficacious, with the exceptions of interpersonal psychotherapy (which was more effective; *d* = 0.20) and nondirective supportive treatment (which was less effective than the other treatments; *d* = −0.13) (8). New avenues to treat major depressive disorders in adults are offered such as ketamine (10), nutritional interventions such as thiamine (11), and omega-3-polyunsaturated fatty acids (12–15) as well as neuromodulation (16).

Following the same profile as the SSRI fluoxetine, animal studies indicated that physical exercise training could be a useful tool in preventing and treating depressive disorders (17). This easy to apply and cheap intervention is an effective intervention against depression (18, 19) with similar effectiveness compared to other forms of treatment in adult humans (20) plus it also provides positive side effects (21).

Adult samples dominate this field of research (22) but age seems to have a significant impact on the effect of exercise on depression (23). Early childhood onset of depression increases chances for depression later in life and has a negative impact on psychosocial development (5, 24, 25). For example, it has been shown that higher order cortical development is dependent on the development of lower order cortical regions (26). We are going to sum up the current literature dealing with the exercise effects on depression in children and adolescents in the following paragraph.

### Characteristics of Depression in Children and Adolescents

The World Health Organization defines adolescence as the stage between 10 and 19 years of age (27). The American Academy of Pediatrics extended this stage to 21 years with the so-called late adolescence (28). This paper follows the extended definition.

Depression is one of the most common mental disorders in children and adolescents. According to a meta-analysis carried out by Costello, Erkanli (1) including studies from around the world, the estimated prevalence in young children (under 13) is 2.8%. Furthermore, 5.6% of all adolescents (13 to 18 years) suffer from depression, and the number of female patients (5.9%) is higher than the number of male patients (4.6%). In the National Comorbidity Replication study it was shown that mental health disorders' onset (like depression) peaks at the age of 14 years (29, 30). According to the WHO, depression and anxiety disorders are among the top five causes of overall disease burden among children and adolescents in Europe (31). Depressive symptoms and clinically relevant depressive disorders in young children can have a huge negative impact on the psychosocial development of the individual and can increase the risk of suicide (5, 25). Suicide is the third most common cause of death in adolescents (2). In general, depression is associated with a shorter lifespan (32).

Although psychological disorders in children and adolescents have not dramatically increased over the past decades for girls there seems to be a significant change in the prevalence of depression compared to earlier decades (33). This sex difference, however, seems not to appear before the end of Tanner stage III, suggesting hormones being involved in the pathophysiology of this disorder (30, 34). The average duration of a major depressive episode in children is 7 to 9 months (35), while 90% of children with depression recover from such an episode (36). In some cases, depression has a chronic progression that reduces the likelihood of therapy success (25). Furthermore, every single depressive episode increases the risk of recurrence (37).

In the treatment of mild and moderate depressive symptoms in children and adolescents, nonpharmacological approaches such as psychotherapy play a major role. A 2006 meta-analysis found modest benefits for psychotherapy versus control (38) which was confirmed by Eckshtain, Kuppens (39) with effects being significantly larger for interpersonal therapy (40) than for cognitive behavioral therapy (CBT). A network meta-analysis (NMA) of youth depression treatment and prevention studies, conducted by Zhou, Hetrick (41), found only CBT and IPT to be significantly more beneficial than most control conditions. A severe symptomatology may demand a combination with antidepressants. A study conducted with adolescents by Foster and Mohler-Kuo (42) found that the combination of cognitive-behavioral therapy and fluoxetine was more effective than drug therapy alone. The SSRI fluoxetine is the first-choice medication for the treatment of juvenile depression. As second-choice antidepressants the SSRIs sertraline, escitalopram, and citalopram might be used (43). Side effects of a pharmacotherapy in adolescents are comparable to those in adults including sedation, agitation, weight gain, sleep problems, vegetative symptoms, and sexual dysfunction (43). Electroconvulsive therapy use in adolescents is considered a highly efficient option for treating depression, achieving high remission rates, and presenting few and relatively benign adverse effects. Risks can be mitigated by the correct use of the technique and are considered minimal when compared to the efficiency of ECT in treating psychopathologies (44).

Preliminary results also suggest repetitive transcranial magnetic stimulation as an effective and well tolerated antidepressant treatment for adolescents with treatment resistant depressive symptomology (45).

### Physical Exercise and its Beneficial Impact on Health

It has been already demonstrated that physical exercise as well as physical activity can cause benefits at both, physical and mental level (46–48). Physical activity interventions have shown to be efficient not only to produce therapeutic benefits when implemented solely or as a part of a treatment for mental disorders, but also to prevent or delay the appearance of mental disorders (49). Additionally, physical exercise was found to be effective in treating symptoms and in reducing the mortality related to major depression (32). As this review discusses, meta-analyses on the depression-reducing effect of exercise in children and adolescents (50–53) so far suggest small to medium effects in this age group. Further moderating variables (e.g., dose specificity) could not be identified so far (51) although study quality and participants' characteristics (e.g. overweights as targets) seem to affect results (50). Several mechanisms have been suggested as responsible for the positive effects of exercise on depression including changes in HPA axis activity, mononamine levels, and neurotrophic growth factors as well as the adaptation of different neural structures [for an overview see Wegner, Helmich (19)].

Along with antidepressant drugs and psychotherapy, physical exercise is a promising option to treat depression. However, in reports exercise is often used synonymously with physical activity (54), which can be misleading. Physical activity is an umbrella term that includes sub-categories such as sports, leisure activities, dance, and physical exercise (55). The American College of Sports Medicine (56) defines physical activity as any bodily movement produced by the contraction of skeletal muscles that results in a substantial increase in caloric requirements over resting energy expenditure. Physical exercise, the concept of interest in this article, is characterised as a training exercise intervention that is planned and structured, repetitive and purposeful, leading to a change in fitness (54). We think that interventions can only run by exercise not by physical activity. Therefore, it can be said that physical exercise is always physical activity, but physical activity is not necessarily physical exercise. Nevertheless, we also included physical activity to our search because the use of exercise as an intervention for the treatment of mental health diseases is still used vaguely (57).

This article aims to evaluate whether children and adolescents with depression benefit in the same way from physical exercise training as adults do. The relief of depressive symptoms in both clinical and nonclinical samples was analysed.

## Materials and Methods

### Protocol and Registration

The protocol of this systematic review was registered on July 20, 2018 in PROSPERO (International prospective register of systematic reviews) at www.crd.york.ac.uk under the PROSPERO-ID CRD42018100357.

### Eligibility Criteria

To determine if a meta-analysis was appropriate for this article the single studies included had to fulfill the eligibility criteria displayed in **Table 1**. In order to structure the eligibility criteria, the PICOS approach (58) was used by implementing five categories: population, intervention, comparator, outcome, and study design. Only meta-analyses, including longitudinal studies with control groups, were considered for inclusion. Their results are usually put in relation to a baseline collected at the beginning of the study, which allows a comprehensive understanding of the degree and direction of change over time (59).

**Table 1:** Eligibility criteria by category (PICOS).

Category	Eligibility criteria
Population	Children and adolescents (≤21 years) with a) a clinical diagnosis of depression diagnosed using clinical recognized diagnostic criteria or b) depression or depressive symptoms assessed using any recognized diagnostic criteria
Intervention	Intervention consisting of physical exercise or physical activity
Comparator	Control group
Outcome	Benefits of physical exercise/activity Effect size reported
Study design	Randomized controlled trials, cluster randomized controlled trials, controlled trials, longitudinal studies

Furthermore, the meta-analyses had to be published in a peer-reviewed journal and in the English language. All limitations were set before the literature search was conducted.

### Search Strategy

A wide literature search strategy was developed using keywords and Medical Subject Headings from four categories: population, outcome, intervention type, and study design (**Supplementary Material Table S1**). The search terms from each category were combined in order to locate all relevant literature using the following databases: PsycINFO (EBSCO Interface), PsycARTICLES (EBSCO Interface), MEDLINE (via PubMed) and PubMed. The search was last conducted December 12, 2019.

### Study Selection

The selection of meta-analyses was carried out independently by two researchers. Any disagreement between them was solved through discussion with a third reviewer. After deleting duplicates, the relevant articles were selected by screening the titles and examining the abstracts. Full-text articles were retrieved and scanned when abstracts did not provide sufficient data.

### Data Extraction

For each of the included meta-analyses the following data was extracted independently by two researchers: Information about the included single studies (design, sample size, sample characteristics, depression assessment, intervention characteristics, control group characteristics) and information about the meta-analyses (risk of bias analysis, effect sizes, methodological characteristics). A data extraction form was used.

### Quality Assessment

All contained meta-analyses performed a risk of bias analysis to assess the quality of the included single studies. Additionally, they focused on publication biases. In order to compare the data, the quality analyses were extracted and examined. To assess the methodological quality of the included systematic reviews with meta-analyses, the AMSTAR 2 checklist (a measurement tool to assess the methodological quality of systematic reviews) (60) was filled out independently by three researchers.

### Effect Size Calculations

In all meta-analyses included, the standardized mean difference (*SMD* = *M_1_* – *M_2_*/*SD_pooled_*) was used as the measure for effect size. The reported effect sizes of each one of the included meta-analyses were combined and a general effect size was calculated and discussed.

### Additional Analysis

A subgroup analysis regarding the effect size for only clinical samples was carried out, understanding clinical samples as those including participants in treatment for a depressive-related disorder or with a formal diagnosis of a depressive disorder. Therefore, the single studies within the selected meta-analyses which examined clinical participants were extracted, the overlap studied, and the effect size calculated. Regarding the assessment of heterogeneity, a visual inspection of the forest plot and the I^2^ value was made. According to the interpretation guide provided by Higgins and Green (61), while I^2^ test results ranging from 0% to 40% might not report relevant heterogeneity levels, results from 30% to 60% may indicate moderate heterogeneity and between 50% and 90% substantial heterogeneity.

Potential publication bias was evaluated using a funnel plot. All statistical analysis and calculations were performed using the Review Manager (RevMan) software (62).

## Results

### Study Selection

**Figure 1:**
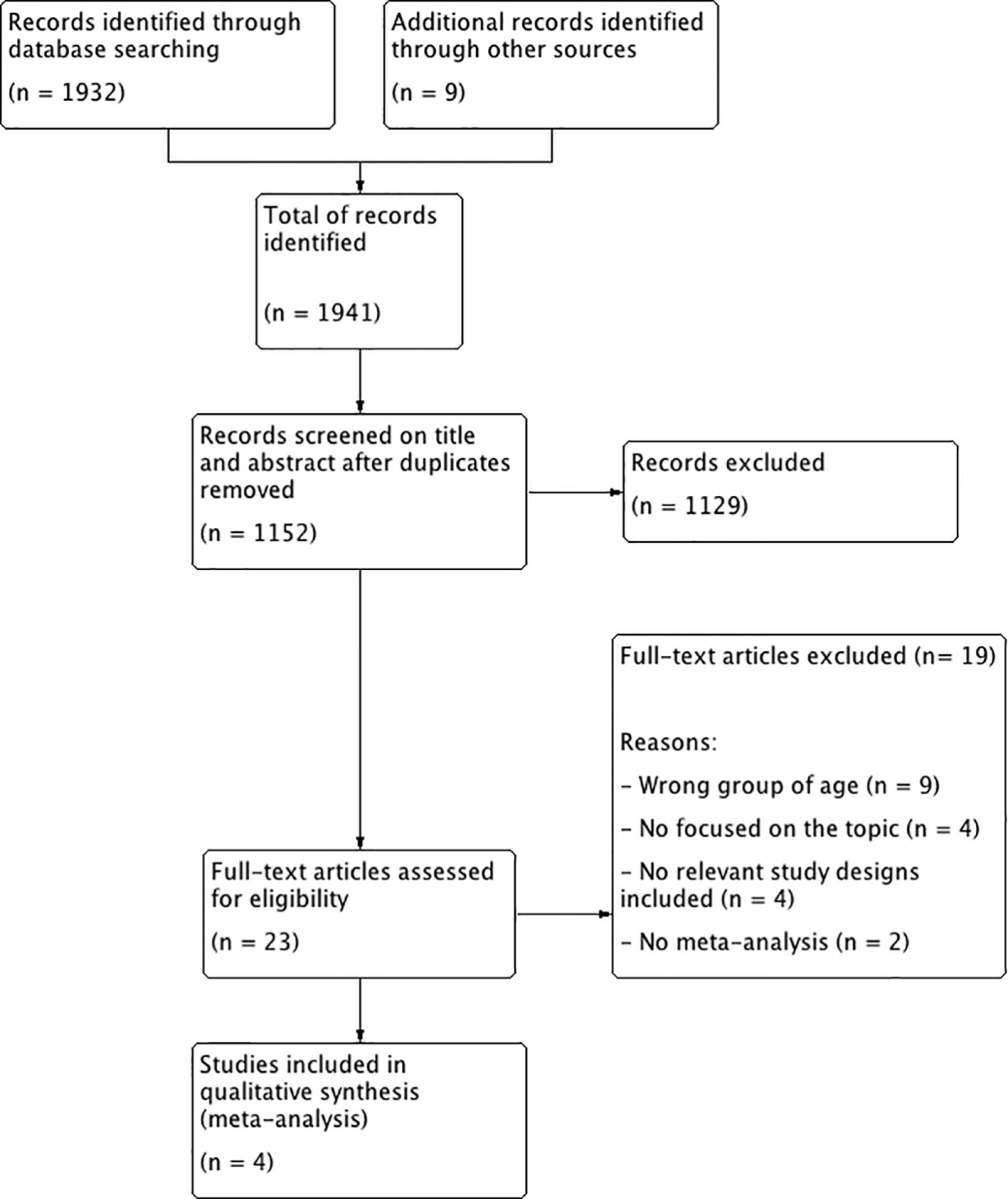
Flow chart of the selection process.

A total of 1,941 studies were identified in the literature search process to seek out systematic reviews with meta-analysis focused in this field. After removing duplicates, two independent researchers reviewed 1,152 titles and abstracts. Any discrepancy between researchers was discussed with a third reviewer. A consensus was reached and ended in a total of 23 potentially relevant studies. Those 23 studies were reviewed in full text. Four studies met the eligibility criteria and were included in this review (50–53) (for more information see the Flow chart of the selection process, **Figure 1**). A table with the excluded studies can be found as **Supplementary Material Table S2**.

### Study Characteristics

The general characteristics information of the four meta-analyses included were extracted by two reviewers using a data extraction form and were summarized in **Table 2**.

**Table 2:** General characteristics of included meta-analyses.

Authors	Studies	Sample	Age	Design	Population	Depression measurement	Intervention	Control group	Effect Size
Larun et al. (52)	16	1,191	11 – 19 years	RCTs	General population; at-risk; in treatment. (with or without CD)	BDI; HADS; RADS; POMS; MAACL; CDI.	Fitness training -walking, running, aerobics- (n=4); weight training (n=1). Length and frequency: From 6 to 40 weeks.	Yes: children on a waiting list, a nonintervention group, a low intensity exercise group or a psychosocial intervention group.	−0.66
Brown et al. (50)	9	581	5 – 19 years	5 RCTs 2 CTs 1 CRCT 1 QES	General population; at-risk for depression; overweigh; criminally institutionalized youth offenders; low socioeconomic status. (without CD)	BDI; CDI; POMS; HADS; SMFQ-SF, MAACL; RCDS.	Aerobic exercise (n=6); health education, sport and physical education lessons (n=2); yoga and mindfulness (n=1). Length and frequency: From 9 to 40 weeks; between 20 and 90 min per session; 2–5 days per week.	Yes; without intervention.	−0.26
Carter et al. (53)	8 (out of 11) are eligible for meta-analysis	445	13 – 17 years	RCTs	General population (n=5); moderate depression from an “at risk” population in a juvenile delinquent institution (n=1); clinical sample (n=5). (with or without CD)	BASC-2; BDI; BYI-II; CBT; CDI; CDRS- R; HADS; MDD; HAMD; SCL-90- R.	Some form of aerobic; resistance; or strength training. Length and frequency: From 6 to 40 weeks; between 15 and 90 min per session; 3 times per week (the majority).	Yes: the usual exercise routine as a control (n=4); equivalent conditions to the intervention group (n=4); no-treatment control condition (n=2); usual psychiatric treatment (n=1).	−0.48
Radovic et al. (51)	8	297	12 – 18 years	5 RCTs 3 CTs	Clinical (n=3) and no clinical (n=5) depressed samples. (with or without CD)	BDI; CDRS- R; CESD; HAMD.	Aerobic exercise (n=6); mixed aerobic exercise and sports training (n=1); mixed aerobic and resistance exercise (n=1). Length and frequency: From 4 to 20 weeks; between 25 and 90 min per session; 2–5 sessions per week.	Yes: a lower dose of exercise group; psychosocial rehabilitation program for young offenders; resistance exercise; no treatment; nutrition sessions; engagement in the regular daily activities in a residential facility.	−0.61

BASC-2, Behavior Assessment System for Children, Second Edition; BDI, Beck Depression Inventory; BYI-II, Beck Youth Inventory Second Edition; CBT, cognitive behavioral therapy; CD, clinical depression; CDI, Children's Depression Inventory; CDRS-R, Children's Depression Rating, Scale Revised; CESD, Center for Epidemiological Studies Depression Scale; CRCT, cluster randomized controlled trial; CTs, controlled trials; HADS, hospital anxiety and depression inventory; HAMD, Hamilton Depression Rating Scale; MAACL, the multiple adjective check list; POMS, profile of mood states; MDD, major depressive disorder; n, number of single studies; QES, quasi-experimental study; RADS, Reynold's adolescent depression scale; RCDS, Reynolds child depression scale; SCL-90-R, Symptom Check List-90-Revision; RCTs, randomized controlled trials; SMFQ-SF, short mood and feelings questionnaire.

### Methodological Characteristics

Of the four meta-analyses included, only two included RCTs (52, 53). The other two meta-analyses included RCTs and also other study designs of lower quality. Radovic, Gordon (51) included five RCTs and three controlled trials (CTs). The meta-analysis of Brown, Pearson (50) covered five RCTs, two CTs, one cluster randomized CT (CRCT) and one quasi-experimental study (QES).

Several questionnaires for reporting depression outcome measures were included in the single studies. The Beck Depression Inventory (BDI) (63) was by far the most frequently used one. The existence of control groups is reported in the four meta-analyses (50–53). Their nature varied between single studies: without intervention, on a waiting list, low intensity exercise, or the usual exercise routine, among others (**Table 2**).

### Intervention Characteristics

The type of intervention differed between single studies. In three of the four included meta-analyses (50–52), aerobic exercise was the most used intervention. Carter, Morres (53) also included articles using some form of aerobic and/or resistance/strength training in the intervention. Within the single studies, the duration of the interventions varied from 4 to 40 weeks. In the meta-analysis of Radovic, Gordon (51), the maximum duration was only 20 weeks, whereas the minimum in Brown, Pearson (50) was at least 9 weeks. The approximated medium duration of all of the interventions was 11.5 weeks.

Regarding the frequency of the interventions, Brown, Pearson (50) and Radovic, Gordon (51) included single studies with a frequency of 2 to 5 days per week. In Carter, Morres (53) and Larun, Nordheim (52), the majority of studies included three sessions per week. In general, the mostly adopted frequency of implementation was three times per week. The duration of the exercise interventions varied between 5 and 90 min per session. The minimum of min was included by Larun, Nordheim (52). Carter, Morres (53) also included single studies with a minimum duration of 15 min only, whereas the other meta-analyses included minimums of 20 or 25 min. The maximum of a 90-min intervention was shared by all meta-analyses. The approximated medium duration of sessions was 41 min.

### Participants Characteristics

The number of participants included in each meta-analysis varied according to the sample size of the single studies. Larun, Nordheim (52) had the largest sample. They included 16 studies and reported 1,191 subjects. The smallest sample was found in Radovic, Gordon (51), with eight studies and 297 subjects. Brown, Pearson (50) integrated nine single studies and a total of 581 subjects. Carter, Morres (53) used eleven single studies, although only eight of them (including 445 subjects) were eligible for their meta-analysis. Those numbers led to a total of 41 single studies and 2,514 subjects as a base for this analysis.

The overlap of single studies within the four analyzed meta-analyses (**Table 3**) caused a reduction to a final number of 30 single studies and 2,110 subjects for this review. The number of participants reported from each meta-analysis regarding the single studies did not always match. Some took the starting sample while others selected the final sample after drop outs. In this article, the latter was reported. This can be seen with the example of a single study (64) which was included in the four selected meta-analyses for this review (50–53). Two of the meta-analyses (51, 52) reported a sample size of 43 participants whereas Carter, Morres (53) reported 60 participants and Brown, Pearson (50) reported 30 participants. In the original article (64) 60 subjects started the program (30 control group; 30 experimental group). However, only 23 subjects of the experimental group and 20 of the control group completed the whole study, giving a final sample of 43 participants.

**Table 3:** Overlap of single studies.

Larun et al. (52)	Brown et al. (50)	Carter et al. (53)	Radovic et al. (51)
Hilyer et al. (64)	Hilyer et al. (64)	Hilyer et al. (64)	Hilyer et al. (64)
Goodrich, 1984	MacMahon & Gross,1987	Kanner, (65)	MacMahon & Gross, 1987
Roth & Holmes, 1987	Norris et al., 1992	Brown et al. (66)	Brown et al. (66)
Berger et al., 1988	Annesi, 2005	Bonhauser et al., 2005	Koniak-Griffin, 1994
Beffert (1994)	Bonhauser et al., 2005	Jeon et al., 2005	Stella et al., 2005
McCann & Holmes, 1984	Daley et al., 2006	Melnyk et al., 2009	Gordon et al., 2010
MacMahon & Gross, 1987	Melnyk et al., 2009	Roshan et al. (67)	Roshan et al. (67)
McArthur & Emes, 1989	Petty et al., 2009	Khalsa et al., 2012	Hughes et al. (68)
Kanner (65)	Mendelson, 2010	Hughes et al. (68)	
Brown et al. (66)		Melnyk et al., 2013	
Cohen-Kahn (69)		Carter et al. (70)	
Lau, 2004			
Smith, 1983			
Bonhauser et al., 2005			
Carl, 1984			
Jacobs, 1984			

The age of the participants ranged between 5 and 20 years. The characteristics of the included populations in each meta-analysis varied from normal population: healthy samples, to at-risk groups: juvenile delinquents, pregnant adolescents, obese children, or clinically depressed populations: with major depressive disorder (MDD), with primary diagnostic of childhood depression and dysthymia or with moderate depressive symptoms.

Three of the included meta-analyses consisted of both clinical and nonclinical samples. Brown, Pearson (50) did not include clinical samples with regards to understanding a clinical sample as the sample with a formal diagnosed depression using clinical recognized diagnostic criteria. Larun, Nordheim (52) and Radovic, Gordon (51) both included three single studies with clinical samples. Carter, Morres (53) included five studies with clinical samples. Due to the overlap of those 11 studies, six single clinical studies were finally identified.

Two of those six final single studies integrated adolescents with diagnosed major depressive disorder (67, 68). One study included adolescents with dysthymia and primary diagnosis of conduct disorder (66). Cohen-Kahn (69) and Kanner (65) included psychiatric inpatients. In the second one, the patients had moderate or severe levels of depression. In Carter, Guo (70), the participants were also receiving a health or social care professional treatment for depression.

### Quality Assessment

#### Quality Assessment of Single Studies

All included meta-analyses conducted a risk of bias analysis regarding the single studies. Three of them (50, 51, 53) used the Delphi method. Larun, Nordheim (52) used another criterion. According to Radovic, Gordon (51), six of their eight articles included were of low quality. One study scored five representing moderate quality and another one scored seven representing high quality, with nine being the highest possible rating. Nevertheless, one item was removed from the original Delphi List in two of the meta-analyses. Brown, Pearson (50) and Carter, Morres (53) removed the care provider blinding item. According to them, in an exercise intervention it is not possible to allow the therapist blinding. Therefore, Carter, Morres (53) considered a study to be of high-quality when scoring five and above. Larun, Nordheim (52) used a different seven-criteria list and analyzed the following items: generation of allocation sequence, concealment of allocation, co-interventions, baseline comparability, intention-to-treat analysis, losses to follow up, and blinding of outcome assessor. None of their included studies were rated as of high quality. The ratings in this case were: for high quality studies needed to fulfill at least six of the criteria; three to five criteria fulfilled equal moderate quality rating; and two or less criteria fulfilled equal a low-quality rating. The overlapping single studies within the four meta-analyses did not necessarily receive the same score in all of the risk of bias evaluations reported by different authors even when using the same assessment tool. This fact reveals the variety of possible interpretations in the valuation with this type of tool.

#### Quality Assessment of Meta-Analyses

The results obtained with the AMSTAR 2 Checklist (60) regarding the methodological quality of the meta-analyses are shown as **Supplementary Material Table S3**. The final agreement between the three independent researchers produced the following results: Larun, Nordheim (52) = moderate quality review; Brown, Pearson (50) = moderate quality review; Carter, Morres (53) = moderate quality review; and Radovic, Gordon (51) = low quality review.

### Synthesis of Results

The aim of this article is to systematically review the meta-analyses that focus on the effects of physical exercise interventions on clinical and nonclinical depression in children and adolescents. Therefore, a first calculation of the effect size reported in the four meta-analyses included was carried out. The overlap was not taken into account at this stage.

Following the interpretation guideline according to Cohen's criteria (small *d* = 0.20; medium *d* = 0.50; large *d* = 0.80) (71), the calculated overall effect size is medium (*d* = −0.50).

### Clinical Samples Analysis

An additional analysis with all of the single studies including clinical samples was carried out. The effect size of the physical exercise intervention in clinical depressive subjects was calculated.

After analyzing the overlap between single studies with clinical samples (**Table 4**), the reported effect sizes were studied. The study of Hughes, Barnes (68) was included in Carter, Morres (53) and Radovic, Gordon (51) reporting different effect sizes. After analyzing the original study, the data of Carter, Morres (53) were used because the same results (*d* = −0.69) were reached. Radovic, Gordon (51) reported an effect size of *d* = −0.54. However, Carter, Morres (53) and Radovic, Gordon (51) showed the same results (*d* = −1.39) regarding the single study of Roshan, Pourasghar (67).

**Table 4:** Overlap of single studies with clinical sample.

Larun et al. (52)	Carter et al. (53)	Radovic et al. (51)
Brown et al. (66)	Brown et al. (66)	Brown et al. (66)
Kanner (65)	Kanner (65)	Hughes et al. (68)
Cohen-Kahn (69)	Hughes et al. (68)	Roshan et al. (67)
	Roshan et al. (67)	
	Carter et al. (70)	

The single study of Brown, Welsh (66) was included in three meta-analyses (51–53). The original article was checked in order to understand the reported data of each of the meta-analyses. Radovic, Gordon (51) reported an effect size of *d* = 0.15. Larun, Nordheim (52) reported an effect size of *d* = 0.78. Carter, Morres (53) explained the impossibility of estimating the effect size due to the insufficient data reported in the original study (standard deviation missing) (53). Due to the missing data, it was agreed upon the impossibility of calculating the effect size.

The study by Cohen-Kahn (69) was included in Larun, Nordheim (52). The effect size reported was *d* = −0.14.

Kanner (65) single study was included in two meta-analyses (52, 53). The selected data was extracted from Larun, Nordheim (52) reporting an effect size of *d* = −0.46. In Carter, Morres (53) two different effect sizes were reported for the single study. The explanation to this fact relapse in the two separate intervention arms—low intensity/high intensity—of the exercise intervention. The reported results were the following: *d* = 0.01 for the first condition and *d* = −0.31 for the second condition.

Another single study (70) was included in the meta-analysis of Carter, Morres (53). The effect size of the exercise intervention with severe depressive participants was *d* = −0.19 in favor of the intervention group.

**Figure 2:**
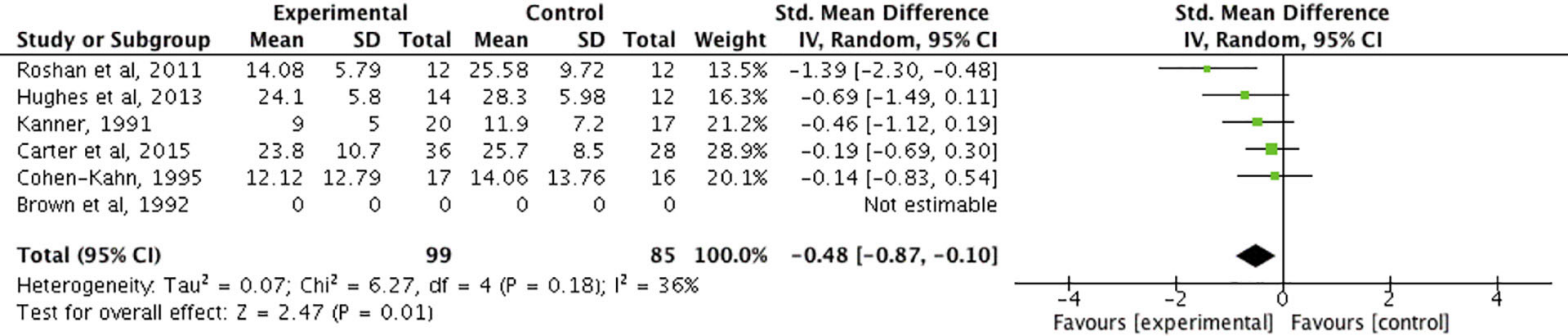
Forest plot of the clinical sample analysis.

The I^2^ statistic test was performed to assess heterogeneity. The results (I^2^ = 36%; *p* = 0.18) showed a moderate level of heterogeneity. Which means that the level of variation across studies is due to the moderate differences between them rather than to chance. The results reported a small to medium effect size taking the base of Cohen's criteria (*d* = −0.48) (71). Effect size calculations for clinical samples can be found in the clinical sample analysis forest plot (**Figure 2**). The publication bias analysis based on the visual inspection of the funnel plot indicated minor to no publication bias due to the asymmetric inverted funnel shape. The funnel plot can be found as **Supplementary Material Figure S1**.

## Discussion

The present article aims to review meta-analyses that focus on the effects of physical exercise on depressive outcome measures in children and adolescents with or without a clinical diagnosis. A medium effect size was found in the general effect size analysis of the included meta-analyses regarding exercise relieving depressive symptoms (*d* = −0.50). This result leads to the conclusion that physical exercise is a promising intervention against depression in the target population. Similar results were found in older individuals where the effect sizes pointing towards the intervention group ranged between moderate (*d* = −0.56) (19) and moderate to large (*d* = −0.68) (72).

The additional analysis of the single studies with clinical samples included in the four meta-analyses selected showed an effect size of *d* = −0.48. According to Cohen's criteria, the effect size is small to medium (71). Similar results were also discovered in clinically depressed adults (73) with an effect size of *d* = −0.40 pointing to the effectiveness of exercise interventions. However, it has been observed that most studies are carried out with nonclinical populations (70). There is a need for more research that includes clinical populations during childhood and adolescence due to the lack of data regarding this specific population.

With these results in mind it can be assumed that physical exercise could be a relevant treatment of depression both in children and adults. These findings are supported by the WHO, who emphasize the psychological benefits of the physical activity in young people with anxiety and depression (47) and make a clear recommendation for the use of physical activity as part of treatment for depressive episodes/disorders in adult populations (74). It should be remembered that physical exercise is included in the physical activity term (55), and is characterized by adding a purpose of achieving changes in fitness following a planned, structured, and repetitive intervention (54).

The most widely used intervention in the four included meta-analyses (50–53) was aerobic exercise. A systematic review carried out with adult samples measured the effects of different interventions on depression. They found no consensus on the correct intensity of aerobic exercise as to achieve the best dose-response relationship (75).

A RCT (76) was carried out to compare the effects of aerobic exercise and antidepressant treatment and showed no differences between groups regarding their level of depressive symptoms after 16 weeks of treatment. This suggests that exercise has the same effectiveness as the standard antidepressant treatments. Nevertheless, the combination of physical exercise with conventional therapies should be looked at more closely and with more effort focused on children and adolescent samples.

The optimal length and frequency of the physical interventions are still a matter of controversy. Dunn, Trivedi (77) examined the optimal dose of exercise needed to improve depressive symptoms in adults with MDD. Their results point to the relevance of higher energy expenditure. One recent publication transports a similar opinion: exercise intensity appears to matter in order to achieve exercise-induced mental health benefits (78). In any case, the following suggestion made by Gronwald, de Bem Alves (79) regarding the exercise intervention prescription seems to be relevant to clarify the real impact of different exercise interventions. They recommend that studies involving physical exercise, or exercise training should be precisely described in detail so that they can be reproduced in other research laboratories, and, more important, can be assessed for their translational impact.

Regarding the methodology, a RCT is the most desirable. The relevance of implementing RCTs to study the effects of interest lies in its quality. This is the most powerful design to determine the existence of cause-effects between intervention and results (80, 81). Therefore, they are widely used for assessing the cost effectiveness of a treatment (82). The importance of controlling for social support when designing the intervention and the need to establish a sham exercise condition has also been highlighted by different authors (20, 83).

In this review, the meta-analyses using only RCTs calculated *d* = −0.66 (52) and *d* = −0.48 (53) as effect sizes. Radovic, Gordon (51) included five RCTs and three CTs and reached an effect size of *d* = −0.61. The effect size reported by Brown, Pearson (50), who also included other study designs, was the smallest (*d* = −0.26). Those authors acknowledged that studies with higher quality ratings showed greater treatment effects. Furthermore, they calculated an effect size of *d* = −0.35 for their included RCTs studies and *d* = −0.14 for the studies with other designs.

## Conclusion

It can be summarized that research reveals small to medium but consistently positive effects of physical exercise on depressive symptoms in clinical and nonclinical samples as well as no negative side effects for children and adolescents. Especially with this last part in mind, physical exercise should be seen as a promising future supplementary intervention against mental health problems in this age group. Therefore, more research in this field is of well-known importance. The lack of literature focused on children and adolescent samples compared to adults, and the responsibility of achieving better life conditions for children and adolescents with depression, should be enough reason to promote research in this field.

Due to the methodological limitations reported by several authors (20, 23) regarding the blinding conditions, the use of sham conditions to blind participants (83), and care providers is recommended. This will ensure high standard quality assessments to measure the effects of exercise and its intensity in randomized CTs. Besides, this might avoid findings that occur through other reasons such as group dynamics.

## Data Availability Statement

The datasets analyzed in this article are not publicly available. Requests to access the datasets should be directed to sandra.amatriain@udc.es.

## Author Contributions 

HB, SA-F, and MW conceptualized and designed the study, drafted the initial manuscript and reviewed and revised the manuscript. SA-F, AK, EM-R, and SM designed the data collection instruments, collected data, carried out the initial analyses and reviewed and revised the manuscript. All authors have read and approved the final version of the manuscript and agree with the order of presentation of the authors.

## Funding

We acknowledge support by the German Research Foundation (DFG) and the Open Access Publication Fund of Humboldt-Universität zu Berlin. SA-F acknowledges the support of the University of A Coruña through the Inditex-UDC Grant Program for research stays.

## Conflict of Interest

The authors declare that the research was conducted in the absence of any commercial or financial relationships that could be construed as a potential conflict of interest.
