# Supplementary material for: Systematic Review of Meta-Analyses: Exercise Effects on Depression in Children and Adolescents
Source: Front Psychiatry. 2020 Mar 6;11:81. doi: 10.3389/fpsyt.2020.00081 (PMC7068196; doi:10.3389/fpsyt.2020.00081)

Table S1 Search strategy.

| **Category** | **Keywords** | **MeSH**^a^ |
| --- | --- | --- |
| Population | (children OR adolescents) | (Child [Mesh] OR Adolescent [Mesh]) |
| Outcome | (depression OR depressive disorder OR depressive symptoms) | (Depression [Mesh] OR Depressive Disorder [Mesh]) |
| Intervention | ((physical activity OR physical exercise) OR (exercise OR sports)) | (Exercise [Mesh] OR Sports [Mesh]) |
| Study design | ((Randomized Controlled Trial OR controlled clinical trial OR longitudinal) | (Randomized Controlled Trial [Publication Type] OR Controlled Clinical Trial [Publication Type]) |

Table S2 Excluded studies and reasons.

| **Study** | **Reason for exclusion** |
| --- | --- |
| Ahn and Fedewa (2011) | Not relevant study design |
| Bailey et al. (2018) | Wrong group of age |
| Bursnall (2014) | No meta-analysis |
| Cairns et al. (2014) | Not relevant study design |
| Calfas and Taylor (1994) | Not relevant study design |
| Craft and Landers (1998) | Wrong group of age |
| Dolle and Schulte-Körne (2014) | Not focused on the topic |
| Fabricatore et al. (2011) | Not focused on the topic |
| Huang et al. (2018) | Wrong group of age and not focused on exercise |
| Korczak et al. (2017) | Not relevant study design and no interventions |
| Krogh et al. (2017) | Wrong group of age |
| Kvam et al. (2016) | Wrong group of age |
| Liu et al. (2015) | Wrong group of age |
| Mala et al. (2012) | Wrong group of age |
| North et al. (1990) | Wrong group of age and not relevant study design |
| Pascoe and Parker (2019) | No meta-analysis |
| Rodriguez-Ayllon et al. (2019) | Not focused on the topic and non-relevant designs included |
| Shore et al. (2017) | Not focused on the topic |
| Yan et al. (2016) | Wrong group of age |

**References**

Ahn, S., and Fedewa, A.L. (2011). A meta-analysis of the relationship between children's physical activity and mental health. *Journal of Pediatric Psychology* 36**,** 385-397.

Bailey, A.P., Hetrick, S.E., Rosenbaum, S., Purcell, R., and Parker, A.G. (2018). Treating depression with physical activity in adolescents and young adults: a systematic review and meta-analysis of randomised controlled trials. *Psychol Med* 48**,** 1068-1083.

Brown, H.E., Pearson, N., Braithwaite, R., Brown, W., and Biddle, S. (2013). Physical activity interventions and depression in children and adolescents : a systematic review and meta-analysis. *Sports Med* 43**,** 195-206.

Bursnall, P. (2014). The relationship between physical activity and depressive symptoms in adolescents: a systematic review. *Worldviews On Evidence-Based Nursing* 11**,** 376-382.

Cairns, K.E., Yap, M.B., Pilkington, P.D., and Jorm, A.F. (2014). Risk and protective factors for depression that adolescents can modify: a systematic review and meta-analysis of longitudinal studies. *J Affect Disord* 169**,** 61-75.

Calfas, K.J., and Taylor, W.C. (1994). Effects of Physical Activity on Psychological Variables in Adolescents. *Pediatric Exercise Science* 6**,** 406-423.

Carter, T., Morres, I.D., Meade, O., and Callaghan, P. (2016). The Effect of Exercise on Depressive Symptoms in Adolescents: A Systematic Review and Meta-Analysis. *J Am Acad Child Adolesc Psychiatry* 55**,** 580-590.

Craft, L., and Landers, D. (1998). The Effect of Exercise on Clinical Depression and Depression Resulting from Mental Illness: A Meta-Analysis. *Journal of Sport and Exercise Psychology* 20**,** 339-357.

Dolle, K., and Schulte-Körne, G. (2014). [Complementary treatment methods for depression in children and adolescents]. *Praxis Der Kinderpsychologie Und Kinderpsychiatrie* 63**,** 237-263.

Fabricatore, A.N., Wadden, T.A., Higginbotham, A.J., Faulconbridge, L.F., Nguyen, A.M., Heymsfield, S.B., and Faith, M.S. (2011). Intentional weight loss and changes in symptoms of depression: a systematic review and meta-analysis. *International Journal Of Obesity (2005)* 35**,** 1363-1376.

Huang, J., Nigatu, Y.T., Smail-Crevier, R., Zhang, X., and Wang, J. (2018). Interventions for common mental health problems among university and college students: A systematic review and meta-analysis of randomized controlled trials. *J Psychiatr Res* 107**,** 1-10.

Korczak, D.J., Madigan, S., and Colasanto, M. (2017). Children's Physical Activity and Depression: A Meta-analysis. *Pediatrics* 139.

Krogh, J., Hjorthøj, C., Speyer, H., Gluud, C., and Nordentoft, M. (2017). Exercise for patients with major depression: a systematic review with meta-analysis and trial sequential analysis. *BMJ Open* 7**,** e014820.

Kvam, S., Kleppe, C.L., Nordhus, I.H., and Hovland, A. (2016). Exercise as a treatment for depression: A meta-analysis. *Journal Of Affective Disorders* 202**,** 67-86.

Larun, L., Nordheim, L.V., Ekeland, E., Hagen, K.B., and Heian, F. (2006). Exercise in prevention and treatment of anxiety and depression among children and young people. *Cochrane Database Syst Rev***,** CD004691.

Liu, X., Clark, J., Siskind, D., Williams, G.M., Byrne, G., Yang, J.L., and Doi, S.A. (2015). A systematic review and meta-analysis of the effects of Qigong and Tai Chi for depressive symptoms. *Complementary Therapies In Medicine* 23**,** 516-534.

Mala, A., Karkou, V., and Meekums, B. (2012). Dance/Movement Therapy (D/MT) for depression: A scoping review. *The Arts in Psychotherapy* 39**,** 287-295.

North, T.C., Mccullagh, P., and Tran, Z.V. (1990). Effect of exercise on depression. *Exerc Sport Sci Rev* 18**,** 379-415.

Pascoe, M.C., and Parker, A.G. (2019). Physical activity and exercise as a universal depression prevention in young people: A narrative review. *Early Interv Psychiatry* 13**,** 733-739.

Radovic, S., Gordon, M.S., and Melvin, G.A. (2017). Should we recommend exercise to adolescents with depressive symptoms? A meta-analysis. *J Paediatr Child Health* 53**,** 214-220.

Rodriguez-Ayllon, M., Cadenas-Sanchez, C., Estevez-Lopez, F., Munoz, N.E., Mora-Gonzalez, J., Migueles, J.H., Molina-Garcia, P., Henriksson, H., Mena-Molina, A., Martinez-Vizcaino, V., Catena, A., Lof, M., Erickson, K.I., Lubans, D.R., Ortega, F.B., and Esteban-Cornejo, I. (2019). Role of Physical Activity and Sedentary Behavior in the Mental Health of Preschoolers, Children and Adolescents: A Systematic Review and Meta-Analysis. *Sports Med* 49**,** 1383-1410.

Shore, L., Toumbourou, J.W., Lewis, A.J., and Kremer, P. (2017). Review: Longitudinal trajectories of child and adolescent depressive symptoms and their predictors – a systematic review and meta‐analysis. *Child and Adolescent Mental Health*.

Yan, S., Jin, Y., Oh, Y., and Choi, Y. (2016). Effect of exercise on depression in university students: a meta-analysis of randomized controlled trials. *The Journal Of Sports Medicine And Physical Fitness* 56**,** 811-816.

Table S3 AMSTAR 2 rating results.

| **Study** | **Researcher 1** | **Researcher 2** | **Researcher 3** | **Final score** |
| --- | --- | --- | --- | --- |
| Larun et al. (2006) | Moderate quality | Moderate quality | Moderate quality | Moderate quality |
| Brown et al. (2013) | Moderate quality | Moderate quality | Moderate quality | Moderate quality |
| Carter et al. (2016) | Moderate quality | Moderate quality | Moderate quality | Moderate quality |
| Radovic et al. (2017) | Low quality | Low quality | Critically low quality | Low quality |

Figure S1 Funnel plot.


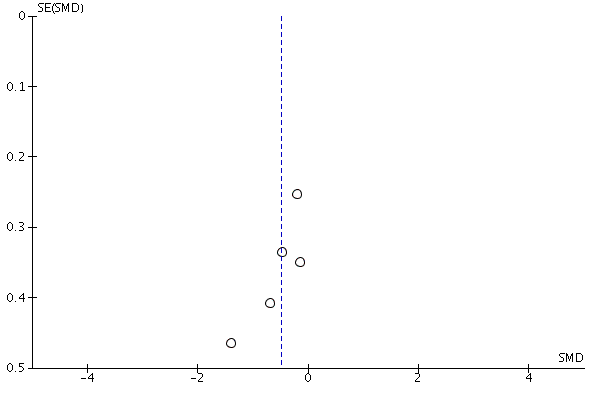

Supplement: Supplementary file 1 [file DataSheet_1.docx]
